# Effect of intramuscular treatment with different iron dextran dosages and non-inferiority study to gleptoferron

**DOI:** 10.1186/s13028-024-00790-6

**Published:** 2025-01-04

**Authors:** Isabel Hennig-Pauka, Martin Ganter, Dirk Bornhorn, Wesley Lyons, Enric Marco, Glen Almond, Bettina Schneider, Lothar Kreienbrock, Ken Steen Pedersen

**Affiliations:** 1 Field Station for Epidemiology, University of Veterinary Medicine Hannover, Foundation, Buescheler Straße 9, D-49456 Bakum, Germany; 2 Clinic for Swine, Small Ruminants, Forensic Medicine and Ambulatory Service, University of Veterinary Medicine Hannover, Foundation, Bischofsholer Damm 15, D-30173 Hannover, Germany; 3 Pharmacosmos Inc., Watchung, NJ 07069 USA; 4 Marco Vetgrup SLP, C. Sant Pauli de Nola 6, 08004 Barcelona, Spain; 5 College of Veterinary Medicine, North Carolina State University, Raleigh, NC USA; 6 Institute for Biometry, Epidemiology and Information Processing, University of Veterinary Medicine Hannover, Foundation, Bünteweg 2, D-30559 Hannover, Germany; 7 Department of Veterinary and Animal Sciences, Faculty of Health and Medical Sciences, University of Copenhagen, Grønnegårdsvej 2, 1870 Frederiksberg C, Denmark; 8 Ø-Vet A/S, Køberupvej 33, 4700 Naestved, Denmark

**Keywords:** Anaemia, Average daily weight gain, Birth weight, Haemoglobin, Packed cell volume

## Abstract

**Background:**

Prevention of iron deficiency in suckling piglets by intramuscular injection of a standardized amount of iron dextran or gleptoferron in the first days of life can lead to over- or underdosage with respective health risks. Currently, combined iron products containing an active substance against coccidia are also used on farms. When using a combination product targeting two diseases, an adjustment of the necessary amount of iron to prevent anaemia in the frame of a farm-specific treatment protocol is not possible. The aim of this study was to test if iron dextran, which can be used in flexible volumes, is statistically non-inferior to a combinatory product, containing gleptoferron and toltrazuril. In addition, different administration schemes for iron dextran with respect to time point and dosage were compared on a conventional farm. Within each out of 17 litters eight healthy piglets were allocated to one of the four treatment groups on the second day of life: (1) 200 mg iron dextran, (2) 200 mg gleptoferron and 45 mg toltrazuril in combination, (3) 300 mg iron dextran, (4) 200 mg iron dextran and additional intramuscular administration of 200 mg iron dextran on day 11 of life. Pigs of groups 1, 3 and 4 received toltrazuril orally. Red blood cell measures were determined prior to treatment on day 2 of life and at weaning. Body weights were measured on day 2, 24, 74 and 160 of life.

**Results:**

Iron dextran was non-inferior compared to gleptoferron within a tolerance range of *±* 5 g haemoglobin/L. In total, treatment groups did not differ with respect to red blood cell parameters and average daily weight gain. The 50% pigs with intermediate birth weights profited from an additional iron dextran administration with respect to higher haemoglobin concentrations at weaning.

**Conclusions:**

In this investigation gleptoferron and iron dextran appear equally appropriate for prevention of iron deficiency anaemia. Piglets of different birth weights might profit differently from an additional iron administration, so that usage of a product containing iron as a single substance is of advantage to allow a flexible adjustment of dosage during the suckling period.

## Background

Iron administration in piglets in their first days of life is a basic zootechnical measure relevant for welfare and health. Dependent on body weight, pigs are marginally equipped with iron at birth ranging from 124 to 180 mg iron/kg dry matter in piglets from 0.8 kg to 1.6 kg body weight [1]. For piglet growth with daily weight gains of 250 g, approximately 10 mg iron/day is needed [2], resulting in iron depletion within the first three weeks of the suckling period without iron supplementation. Iron concentrations of 1.7–5.4 µg/mL in sow colostrum and 1.3–4.6 µg/mL in milk are not adequate to fill this supply gap [3]. Although study outcomes about oral iron supplementation vary with respect to efficacy [4] oral iron uptake from external sources is assessed to be insufficient due to low intestinal iron absorption [5]. The immaturity of the duodenal mechanisms of iron absorption was found to be related to low expression of iron transporters (duodenal divalent metal transporter (DMT) 1 protein, ferroportin) and a high expression of hepcidin as the major iron-regulatory hormone during the first days of life [5]. A physiological tight control of iron metabolisms and regulation of iron release into extracellular body compartments is essential to prevent toxic effects of iron [6]. It can be assumed, that breeding success with regard to high piglet growth rates and high number of piglets born alive overstrain genetically determined and evolutionary meaningful iron homeostasis control mechanisms. The naturally low foetal iron reserves are still low in litters of hyperprolific sows, while in parallel growth rate and feed efficacy of piglets have increased, and also the demand for essential nutrients as iron [7]. While in adults iron homeostasis is dependent on absorption in the duodenum and its recycling by macrophages, iron deficiency anaemia (IDA) in piglets with the consequence of growth retardation, depression and a disturbance of cognitive development can only be prevented by iron supplementation [8, 9].

Immediately after birth a haemodilution caused by colostrum uptake and subsequent shifts in body fluid to the circulatory system leads to low haemoglobin (Hb) concentrations in piglets. The heaviest piglets are considered to be at higher risk to become anaemic due to their larger blood volume [10]. If not treated, a latent iron deficiency in the first living days will finally result in a microcytic hypochromic anaemia characterized by Hb concentrations below 90 g/L and small erythrocytes [11]. Cut-off Hb concentrations considered critical for piglet health differ among experts and are still under discussion. This might be due to a lack of currently valid values for Hb corrected for breed, production phase and age [11]. Some authors suggest a Hb of 110 g/L to exclude latent iron deficiency [7, 10, 12], which corresponds to the global cut-off for children younger than 5 years defined in 1968 in a WHO technical report [13]. In swine, either Hb concentrations below 80 g/L are considered critical for piglet health [14] or Hb concentrations below 90 g/L [9, 10]. Some authors recommended for diagnostic of IDA the determination of iron-related parameters (e.g. ferritin, transferrin and transferrin saturation, iron, total iron binding capacity) next to established haematological and reticulocyte parameters [7, 15–17]. Iron-related parameters are more difficult to interpret with respect to health status, because iron homeostasis is tightly regulated.

Due to the biological duality of iron, balancing of risks and benefits of iron administration in suckling piglets is under research in different production systems with varying available iron sources from feed, water and environment. A comparison of piglets raised in intensive indoor and outdoor production systems and supplemented with 100 mg iron dextran on day three of age resulted in higher Hb concentrations on day 28 of life in outdoor pigs [18]. Some authors conclude that no prophylactic iron treatment is necessary in outdoor pig production [19]. A comparison of organic piglets either raised indoor with iron supplementation or outdoor without iron supplementation resulted in higher Hb concentrations in outdoor pigs, because soil contained sufficient bioavailable iron [20]. In outdoor pigs with physiologically low Hb concentrations on their third day of life and receiving no iron treatment the majority of piglets was not anaemic at weaning [21]. Treated pigs showed lower Hb levels at weaning, indicating reduced iron absorption from the duodenum due to downregulation of ferroportin [21]. Other authors conclude that iron administration in general is necessary in outdoor pig production [22]. In organic farms with prolonged suckling periods one iron injection was found to be not sufficient in spite of iron uptake from external sources as soil and piglet starter diet [22, 23]. Contradictory results might be explainable by differences in bioavailable iron in soil or by other factors as genotype of the sow or season [18, 21].

In intensive pig production daily iron requirements of suckling piglets are usually complied by subcutaneous or intramuscular (IM) administration of iron dextran or gleptoferron in the first days of life [7]. Currently, combined iron products containing an active substance against coccidia are also used on farms. The advantage of these products combining two routine measures in one is that they save time and effort. The parenteral application of a coccidiostat is more comfortable than the formerly established oral application with same efficacy [24]. However, when using a combination product targeting two diseases, an adjustment of the necessary amount of iron to prevent anaemia in the frame of a farm-specific treatment protocol is not possible. The aim of this study was to test for non-inferiority of iron dextran (Uniferon^®^, Pharmacosmos A/S, Holbaek, Denmark), which can be used in flexible volumes, versus to the combinatory product Forceris^®^ (Forceris^®^, Ceva Santé Animale, Libourne, France) containing gleptoferron and toltrazuril. In addition, different administration schemes for iron dextran with respect to time point and dosage were compared on a conventional farm.

## Methods

### Study farm

The animal experiment was performed in a farrow-to-finish farm with 230 sows (Danbred genetics) and 2550 fattening places in a swine dense region in North-Western Germany. Farrowing intervals were five weeks with a four-week suckling period and farrowing groups of approximately 57 sows. Sows of one farrowing group farrowed in three farrowing units with 20 places each. No nurse sows were used, and split suckling was not performed. The farm was known positive but stable for porcine reproductive and respiratory syndrome virus (PRRSV) according to category II-vx [25] and for *Actinobacillus* (*A.*) *pleuropneumoniae* and *Mycoplasma (M.) hyopneumoniae*. No disease outbreaks were observed in the year before start of the study. All sows were vaccinated every four months with a live attenuated PRRSV-1 vaccine (Suvaxy^®^ PRRS MLV, Zoetis Deutschland GmbH, Berlin, Germany) and against influenza virus with a triple vaccine containing H1N1, H1N2 and H1N3 (Respiporc FLU3, Ceva, Düsseldorf, Germany) as well as a vaccine against H1panN1 (Respiporc FLUpanH1N1, Ceva). Sows were further vaccinated every six months against parvovirus and erysipelas (Parvoruvac^®^, Ceva). Piglets are vaccinated routinely against edema disease on day five of life (Ecoporc SHIGA, Ceva) and on day 23 of life against PRRSV-1 (Suvaxy^®^ PRRS MLV), porcine circovirus 2 (PCV2) and *M*. *hyopneumoniae* in combination (Porcilis^®^ PCV M Hyo, MSD Tiergesundheit, Unterschleißheim, Germany). In addition, piglets were vaccinated twice on day 23 and three weeks later with an autogenous vaccine containing killed *Streptococcus* (*S*.) *suis* and *A. pleuropneumoniae* strains. On average 16.7 live and 1.8 dead born piglets with 15% suckling piglet losses were recorded on the farm in the study year. The piglet´s average weights at weaning were 7 kg and the average daily weight gain in the fattening period 0.9 kg.

Study piglets were selected randomly and stratified by weight and sex out of 17 litters of sows in different farrowing units, which had farrowed on the same day. A total number of 136 piglets out of the 17 litters were included in the study on their second day of life. Sows farrowed in conventional crates on a fully slatted floor. Farrowing was not induced medically, supervised on the main farrowing days from 6am to 10pm and assisted if necessary. The ambient temperature in the farrowing room during farrowing was 26 °C due to warm weather conditions in summer. Piglets had access to a water-heated resting area with a surface temperature of 33 °C. No cross-fostering was performed during the experiment in the 17 litters. In all farrowing pens an automatic replacement milk system was implemented to provide milk supplement from day 5 to 11 of life. Creep feeding started at day 12 of life ad libitum with a supplementary feed as training mash (wet creep feed) provided via the automatic replacement milk system until weaning. Since day 16 of life additional dry creep feed was provided in an extra trough ad libitum until three days after weaning. Since day 3 after weaning creep feeding was paralleled by feeding nursery starter diet ad libitum (Table 1). At weaning on day 26 of life piglets were brought litter-wise to the nursery pens with three litters stalled in one pen with place for 33 piglets on fully slatted plastic floor. Pens were equipped with automatic dry feeders with eight feeding places and four nipple drinkers. In addition temporary drinking trays were provided as well as three chains with wooden beams as enrichment objects. Lightweight piglets were collected from different litters and commingled in separate nursery pens. Ambient temperature in the nursery unit was 30 °C for two days at the beginning of the nursery period and decreased to 28 °C until end of the first week. In the following six weeks temperature was decreased by 1 °C to 22 °C until end of nursery. After a seven-week nursery period, pigs were brought to the fattening unit in a neighbouring building with place for ten pigs per pen on fully slatted floors. Water was used from a well and feed for pigs of all production stages was bought from a commercial feed company. Analysis of drinking water showed low nitrate (127 mg/L), low sulphate (107 mg/L) and low iron (< 0.1 mg/L) concentrations, and low number of colony forming units (CFU) of 33–49 CFU/mL after incubation at 22 °C and 17–39 CFU/mL after incubation at 36 °C without any detection of coliforms. Feed iron contents are recorded in Table 1.

Husbandry conditions followed the national German regulations on pig husbandry [26].

**Table 1:** Declarations of iron content in commercial feed used on the farm for different production stages

	Milk supplement	Wet creep feed	Creep feed	Nursery starter diet	Nursery diet	Fattening
**Feeding period (living days)**	5–11	12–28	16–32	32–46	47–80	81–240
**Iron content (mg/kg)**	75	175	106	140	140	not declared
**Substance**	Fe(II)sulfate-monohydrate		Fe(II)sulfate-monohydrate, Fe (II)-Glycin-chelat-Hydrat	Fe(II)sulfate-monohydrate		
**Recommendation for iron content (mg/kg)**	150^1^	240^2^ 175^3^	240^2^	90-150^2^ 100–120^4^	90-150^2^ 100-120^4^	50–60^4^

^1^ [27], ^2^ reviewed by [7], ^3^ [28], ^4^ [29]

### Study design

From the current farrowing group only litters from those sows were included in the study, which had farrowed on the same main farrowing day. Out of this subgroup of 34 sows 17 sows with at least ten and less than 18 live born piglets were selected. In the study group 88% of the sows were in parity 4–7, one sow had the first and one the ninth litter. Within each of the 17 litters eight healthy piglets (four males and four females if available) weighing at least 1 kg were allocated to four treatment groups by target randomization based on weight and sex strata resulting in two piglets per group in each litter, which were sampled and treated according to Table 2. For intramuscular (IM) treatment disposable syringes with a maximum volume of 2 mL (HSW HENKE-JECT^®^, Henke Sass Wolf, Tuttlingen, Germany) connected to disposable needles (20-gauge, 0.90 × 40 mm (Sterikan^®^, B.Braun, Vet Care GmbH, Tuttlingen, Germany) were used. Needles were only used once. Piglets were examined, sampled and weighed at start of the study on day 2 of life, on day 11 of life, at weaning, end of nursery and end of fattening. Ear tagging was performed on day 2 of life. On day 3 of life piglets were tail-docked and on day 6 male piglets were castrated after pain treatment with 0.4 mg meloxicam (Metacam^®^, Boehringer Ingelheim Vetmedica GmbH, Ingelheim, Germany)/kg body weight (BW) and under general anaesthesia by IM injection of 25 mg ketamine (CP-Pharma Handelsgesellschaft mbH, Burgdorf, Germany)/kg BW and 2 mg azaperon (Stresnil^®^, Elanco, Kiel Germany)/kg BW. No further zootechnical measures or treatments were performed routinely and no treatment was necessary in piglets of the study group.

While piglets of all four groups were treated on day 2 of life, only piglets of group 4 were treated again on day 11 of life. All pigs were weighed and sampled again two days before weaning on day 24 of life, weighed again on day 74 of life at the end of nursery and on day 160 of life at the end of fattening.

Study design was elaborated to prove (i) non-inferiority of IM administration of 200 mg iron dextran (1 mL Uniferon^®^) compared to IM administration of 200 mg iron (III)-gleptoferron and 45 mg toltrazuril (1.5 mL Forceris^®^, Ceva Santé Animale, Libourne, France) with respect to the major target variable Hb measured prior to weaning on day 24 of life and (ii) to test if additional amount of iron given on day 2 of life or a repeated iron administration on day 11 of life were beneficial for growth and Hb concentrations. An effect of toltrazuril on gut microflora and therefore indirect on intestinal iron absorption cannot be excluded, so that toltrazuril was given in all experimental groups [30]. Toltrazuril as one component in the combinatory product Forceris^®^ was orally administered as a singular product (Cevazuril^®^, Ceva Santé Animale, Libourne, France) in groups 1, 3 and 4. Similar approaches have been performed previously in other comparative studies [31]. For oral administration a disposable syringe with a maximum volume of 2 mL (HSW HENKE-JECT^®^) were used. The syringe was placed laterally through the piglet`s mouth cleft behind the tongue ground before 0.9 ml Cevazuril^®^ was applied. The piglet’s mouth was closed with gentle pressure until the product was swallowed.

**Table 2:** Experimental groups of piglets provided with different iron treatment strategies

Group	Treatment	Sample size (*n*)
**1**	Single products: 200 mg iron dextran IM, oral application of 45 mg toltrazuril on day 2	34
**2**	Combinatory product: 200 mg gleptoferron and 45 mg toltrazuril IM on day 2	34
**3**	Single products: 300 mg iron dextran IM, oral application of 45 mg toltrazuril on day 2	34
**4**	Single products: 200 mg iron dextran IM, oral application of 45 mg toltrazuril on day 2 and 200 mg iron dextran IM on day 11	34

Within each of 17 litters eight healthy piglets were allocated to the four treatment groups by target randomization based on weight and sex strata resulting in two piglets per group in each litter

### Clinical examination and blood analyses

During the suckling period clinical examination and scoring of skin lesions dorsal on carpal joints and head, claw lesions and navel were performed on day 2, 11 and 24 of life. At the end of nursery on day 74 and at the end of fattening on day 160 of life again a clinical examination and scoring of tail and ear lesions were performed. Clinical variables and their scores are summarized in Table 3.

Blood samples were collected from the *Vena cava cranialis* in volumes of 2 mL in collection tubes containing ethylenediaminetetraacetic acid (EDTA) (Kabevette^®^, Kabe Labortechnik, Mümbrecht-Elsenroth, Germany) using 21-gauge, 0.80 × 40 mm needles (Sterikan^®^, B.Braun). Blood samples were placed in an isolated box with cool pads until end of sampling, transported to the laboratory at the Clinic for Swine and Small Ruminants, University of Veterinary Medicine, Hannover, stored at 6 °C overnight and analysed within 24 h. Haematological variables (Hb, packed cell volume (PCV), erythrocytes, mean corpuscular haemoglobin concentration (MCHC), leucocyte count, thrombocyte count) were automatically analysed in a haematology analyser (Celltac MEK-6550, Nihon Kohden Europe GmbH, Rosbach, Germany).

**Table 3:** Clinical variables recorded on days 2, 11, 24, 74 and 160 of life

Variables	Clinical scores					
	0	1	2	3	4	5
Claws	No claw lesions	Pododermatitis haemorrhagica	Panaritium	-	-	-
Skin lesions head	No skin lesions	Slight and superficial scratches	Dominant red scratches	Wet wounds	-	-
Skin dorsal on carpal joints	No skin lesions	Slight and superficial scratches and crusts	Extended crusts	Wet wounds	-	-
Navel^1^	Physiological diameter and consistency	Diameter 0.8–1.5 cm	Diameter > 1.5 cm	Wet navel	Purulent secretion or abscess	Hernia umbilicalis
Diarrhoea	No	Pulpy feces	Liquid feces	-	-	-
Ear	Intact skin	Dry scratches	Bloody wound	-	-	-
Tail lesion	Intact skin	Dry scratches	Bloody wound	-	-	-
Tail losses	Length after tail docking	*≥* half length of tail	*≥* two third of tail	Total tail loss	-	-

Clinical scores were used in suckling piglets for skin lesions dorsal on carpal joints and head, claw lesions and navel. In nursery and fattening pigs tail and ear lesions were scored. All pigs were examined for any other deviations from physiological findings

^1^only suckling piglets

### Sample size calculation for a non-inferiority study and statistical evaluation

A sample size calculation for a non-inferiority t-test between experimental groups 1 (200 mg iron dextran) and 2 (200 mg gleptoferron) was performed using NCSS PASS (version 2021, v21.03, East Kaysville, Utah, USA) with target power (1-β) 0.8 and target significance level α of 0.05. The calculation was based on a non-inferiority assumption according to expected Hb concentrations as the major target variable deduced from results in three published comparison trials [32–34]. According to these studies a difference of 5 g Hb/L between treatment groups was considered equivalent and defined as a margin for non-inferiority testing between different schemes of iron administration in this study (Table 2).

Sample size calculation resulted in 29 piglets per group, i.e. in total 116 piglets. Due to an average suckling piglet mortality of 15% in the trial farm, the required number of piglets was adjusted by five reserve animals per group resulting in 136 piglets to be included in trial.

Data were recorded in Excel, version 2016 (Microsoft Corporation, Albuquerque, USA) and imported for statistical evaluations into SAS^®^, version 9.4 TS level 1M5 (SAS Institute, NC, USA).

Spearman`s rank correlation coefficients were calculated for all variables with a quantitative outcome. Frequencies of clinical findings were compared by chi-square testing between the groups.

For the main primary hypothesis under study non-inferiority testing was performed to prove that treatment of piglets in group 1 (200 mg iron dextran) is non-inferior to treatment of piglets in group 2 (gleptoferron) with respect to Hb at weaning. A boundary threshold of 5 g/L Hb was chosen for this test (see above). Procedure of non-inferiority testing was based on Schuirmann’s method of two one-sided tests (TOST).

Within explorative analyses for secondary endpoints group differences with respect to red blood cell measurements and weight gain were tested in an analysis of variance model followed by least square means tests for group effects. Due to the explorative nature of these analyses no multiple adjustments were conducted.

In addition, it was explored if treatment effects were influenced by birth weights. To include the potential effects of birth weight in the model, piglets were allocated to three birth weight classes. Three weight groups were defined, i.e. “low weight piglets” from the minimum to the 25% quantile, “medium weight piglets” from the 25% to the 75% quantile, “high weight piglets” from the 75% quantile to the maximum. Allocation of animals by birth weight class and treatment to the groups are shown in Table 4. General linear models with fixed effects group and birth weight class and the combined effects of both were analysed with respect to red blood cell variables at weaning.

**Table 4:** Number of piglets in experimental groups and range of body weights

	Experimental groups							
	Group 1		Group 2		Group 3		Group 4	
**Weight classes**	Body weight (kg)	n	Body weight (kg)	n	Body weight (kg)	n	Body weight (kg)	n
Weight class 1 (low)	1.14–1.48	9	1.12–1.48	7	1.26–1.46	9	1.10–1.48	10
Weight class 2 (medium)	1.52–1.94	16	1.50–1.94	20	1.50–1.94	14	1.56–1.94	14
Weight class 3 (high)	1.98–2.20	8	2.02–2.30	7	1.96–2.46	8	1.96–2.36	9

Number of piglets allocated in experimental groups and weight classes: sample size and range of weights at study start on day 2 of life, n: number of piglets within weight class

## Results

### General clinical and laboratory findings

The 17 sows included in the study had in average 15.6 live born, 8.9 dead born and 14.4 weaned piglets. The suckling piglet mortality was 7.5% in the litters of the study group. During the suckling period four animals out of the study groups were euthanized and sent for necropsy. Due to chronic disease (starvation, arthritis, ascites) these pigs were considered as biological outliers and were excluded from the final statistical examination. One additional pig with severe arthritis was excluded from the study. All injection sites were inconspicuous in all animals during the whole course of the study. In the majority of suckling piglets, a slight pododermatitis haemorrhagica (reddening of the heels) was found, while only one animal showed a panaritium (swelling, reddening and pain sensitivity of the coronary skin) on day 24 of life. Head skin lesions were mainly superficial scratches on day 2 of life. About 30% of the animals showed more severe head skin lesions (score 2 and 3) on day 11 of life. Skin lesions dorsal on carpal joints were as well most severe on day 11 of life with 7.6% of pigs showing open wounds. Most dorsal carpal skin wounds were superficial. Navel inflammations decreased during the suckling period (Table 5). Two pigs were diagnosed with umbilical hernia. A severe purulent inflammation of the navel was found on day 11 in three animals. The incidence of diarrhoea was very low during the whole suckling period and was not treated. During nursery a severe outbreak of disease caused by *S*. *suis* led to animal losses. During the second half of the nursery period a severe tail biting outbreak occurred. In total, due to animal losses and loss of ear tags, 42 animals were excluded from further evaluation. Among the remaining 89 pigs at the end of nursery tail injuries were found in more than half of the pigs. Due to ear tag losses only 76 pigs were identified and examined at the end of fattening. Acute tail wounds were diagnosed still in 5.3% of pigs at the end of fattening and 6.6% of the pigs had a total loss of tail. A summary of clinical findings is shown in Table 5. There were no differences in frequencies of various clinical findings between different treatment groups.

**Table 5:** Selected clinical findings in all examined pigs (%) in the respective stage of production

Proportion of pigs with clinical findings (%)	Day of life				
	2 (*n* = 131)	11 (*n* = 131)	24 (*n* = 131)	74 (*n* = 89)	160 (*n* = 76)
Pododermatitis haemorrhagica	64.9	66.4	3.1	-	-
Head skin lesions (score 2 and 3)	0.8	30.5	5.3	-	-
Dorsal carpal joint skin lesions (score 2 and 3)	0.0	36.6	58.0	-	-
Navel inflammation	27.5	24.4	14.5	-	-
Diarrhoea	3.8	1.5	0.8	-	-
Tail injuries	-	-	-	55.1	5.3
Tail losses	-	-	-	-	14.5
Bloody ear injuries	-	-	-	18.0	0.0

Selected clinical findings in % from all examined pigs (n) in the respective stage of production until end of fattening irrespective of treatment group

Piglets included in the study had a relatively high average birth weight of 1.7 kg and an adequate average daily weight gain of 0.460 kg during the nursery period. Growth rates during fattening were in average 0.926 kg resulting in average heavy weights at slaughter of 111 kg in the study pigs. Body weight development in the different treatment groups is shown in Table 6.

**Table 6:** Body weight and average daily weight gain (ADWG) in different treatment groups (mean *±* standard deviation)

	Group 1		Group 2		Group 3		Group 4	
Day of life	n	Body weight (kg)	n	Body weight (kg)	n	Body weight (kg)	n	Body weight (kg)
2	33	1.71 *±* 0.32	34	1.72 *±* 0.3	31	1.71 *±* 1.86	33	1.69 *±* 0.34
24	33	6.85 *±* 1.45	34	6.62 *±* 1.57	31	6.61 *±* 1.86	33	6.61 *±* 1.81
74	23	31.17 *±* 6.36	23	30.15 *±* 5.57	20	29.78 *±* 5.92	23	29.02 *±* 7.52
160	17	116.82 *±* 12.65	20	110.13 *±* 20.07	19	109.29 *±* 14.99	20	108.23 *±* 22.65
	Group 1		Group 2		Group 3		Group 4	
	n	ADWG (kg)	n	ADWG (kg)	n	ADWG (kg)	n	ADWG (kg)
Suckling period (day 2–24)								
	33	0.23 *±* 0.06	34	0.22 *±* 0.07	31	0.22 *±* 0.07	33	0.22 *±* 0.08
Nursery period (day 24-day 74)								
	23	0.50 *±* 0.12	23	0.48 *±* 0.10	20	0.47 *±* 0.10	23	0.46 *±* 0.13
Fattening period (day 74–160)								
	17	0.97 + 0.19	20	0.91 + 0.19	19	0.92 + 0.13	20	0.91 + 0.2
Suckling, nursery and fattening period (day 2-160)								
	17	0.73 *±* 0.08	20	0.68 *±* 0.19	19	0.68 *±* 0.09	20	0.67 *±* 0.14

**Fig. 1:**
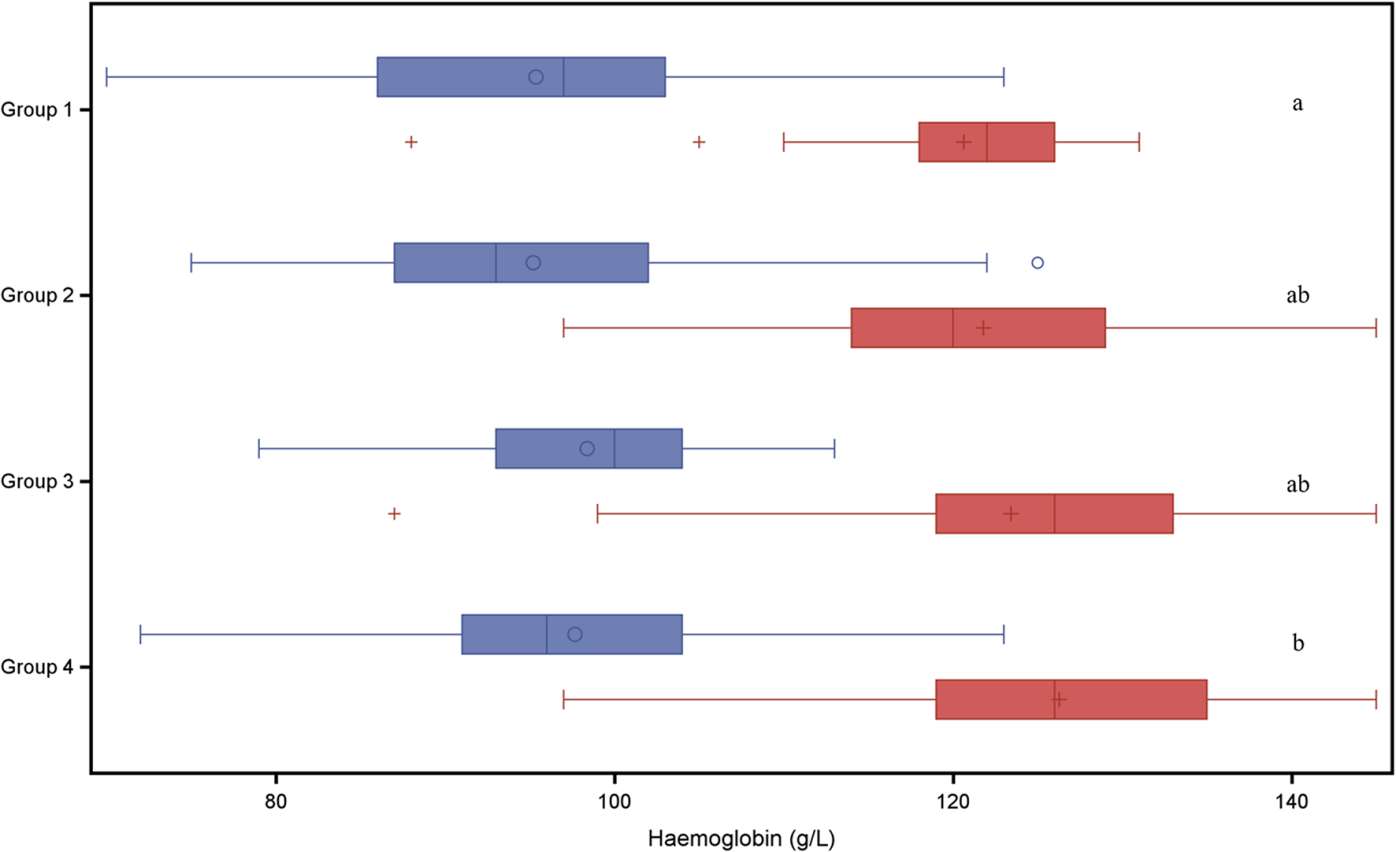
Haemoglobin concentrations (g/L) in piglets belonging to different treatment groups and day of life Blue boxes: 2nd day of life, study start, red boxes: 24th day of life at weaning; different letters right to the boxes indicate significant differences between respective groups 1 and 4 (*P* = 0.04) in a least square means comparison

**Fig. 2:**
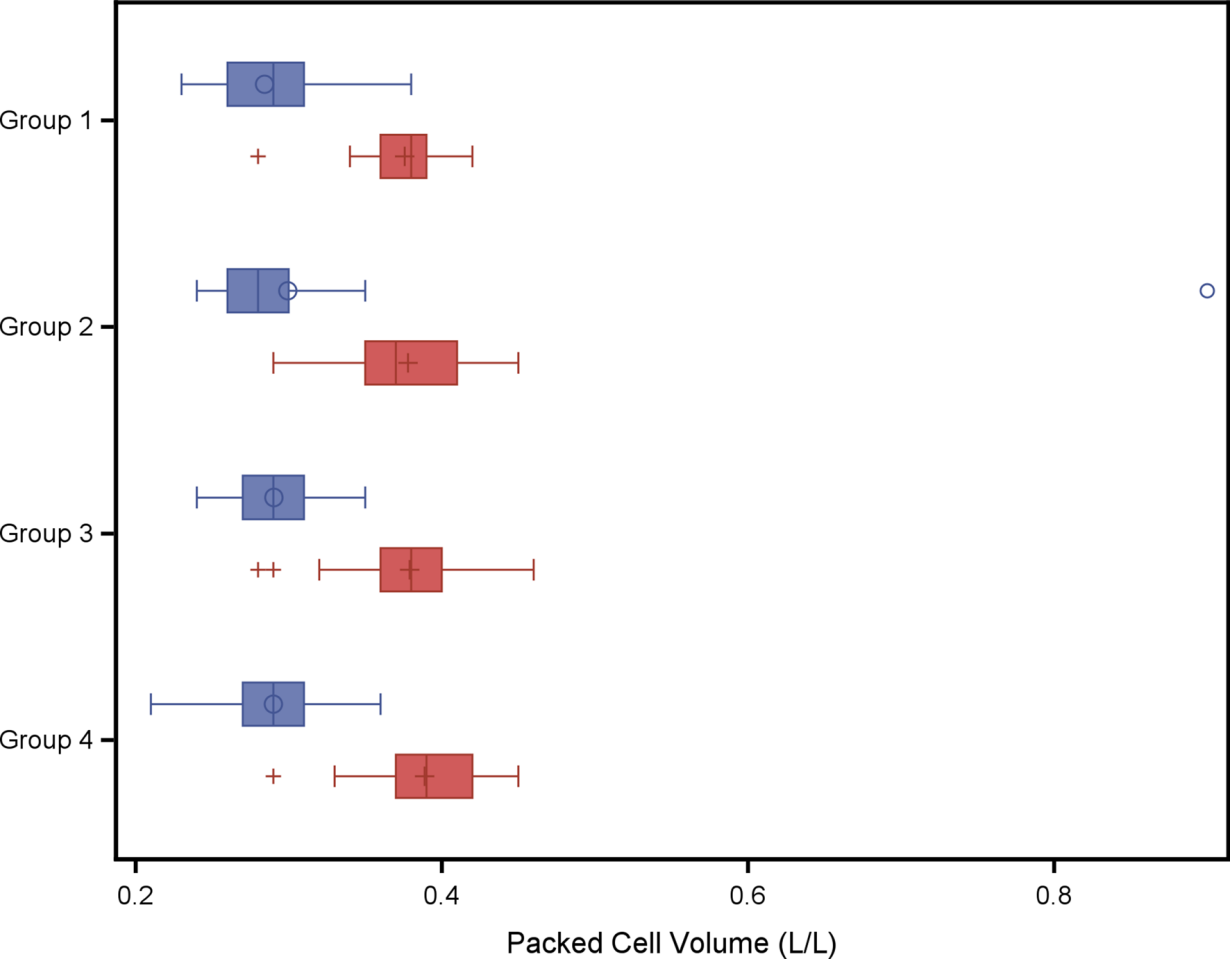
Packed cell volume (L/L) in piglets belonging to different treatment groups and day of life. Blue boxes: 2nd day of life, study start, red boxes: 24th day of life at weaning

**Fig. 3:**
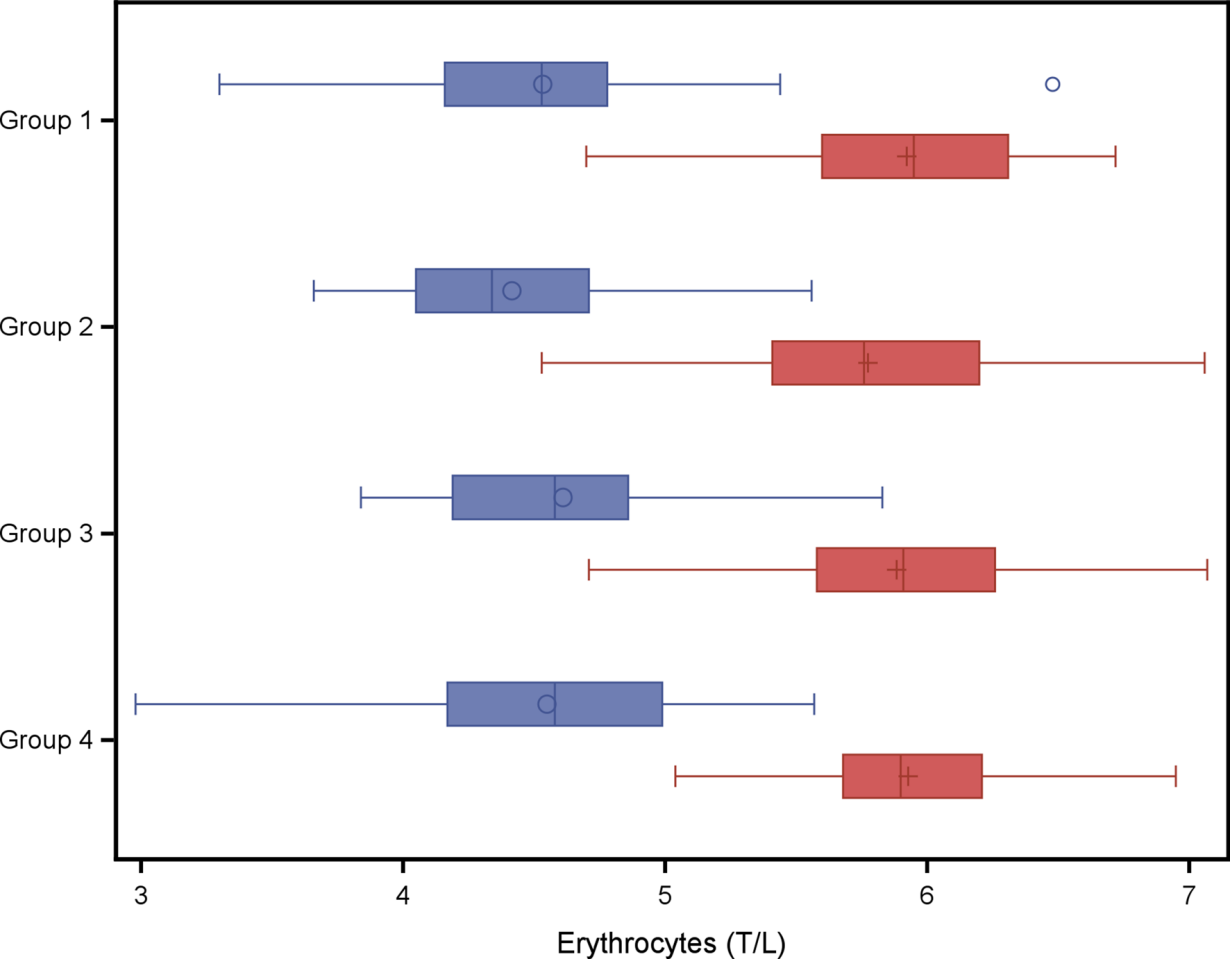
Erythrocytes (T/L) in piglets belonging to different treatment groups and day of life. Blue boxes: 2nd day of life, study start, red boxes: 24th day of life at weaning

**Fig. 4:**
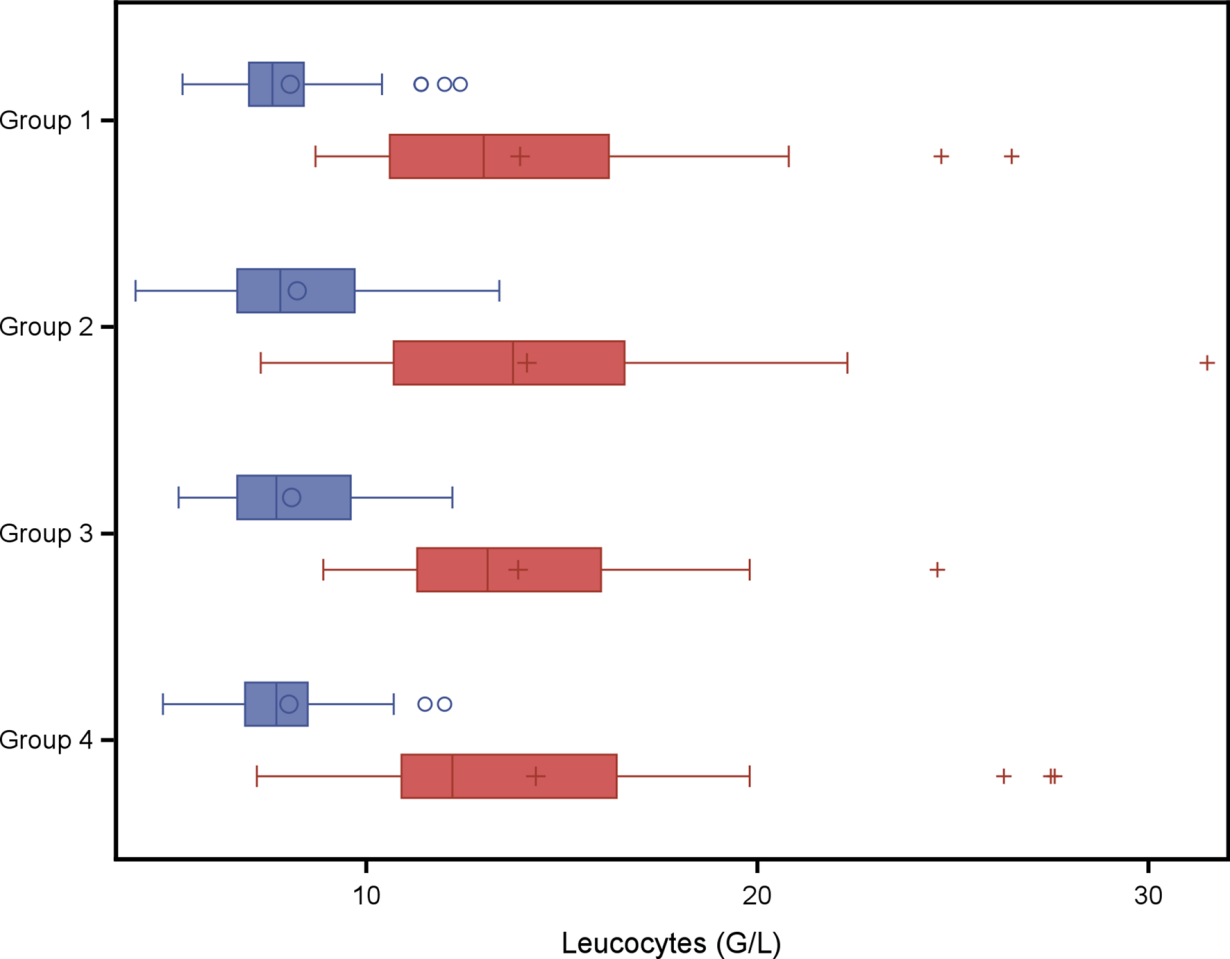
Leucocytes (G/L) in piglets belonging to different treatment groups and day of life. Blue boxes: 2nd day of life, study start, red boxes: 24th day of life at weaning

**Fig. 5:**
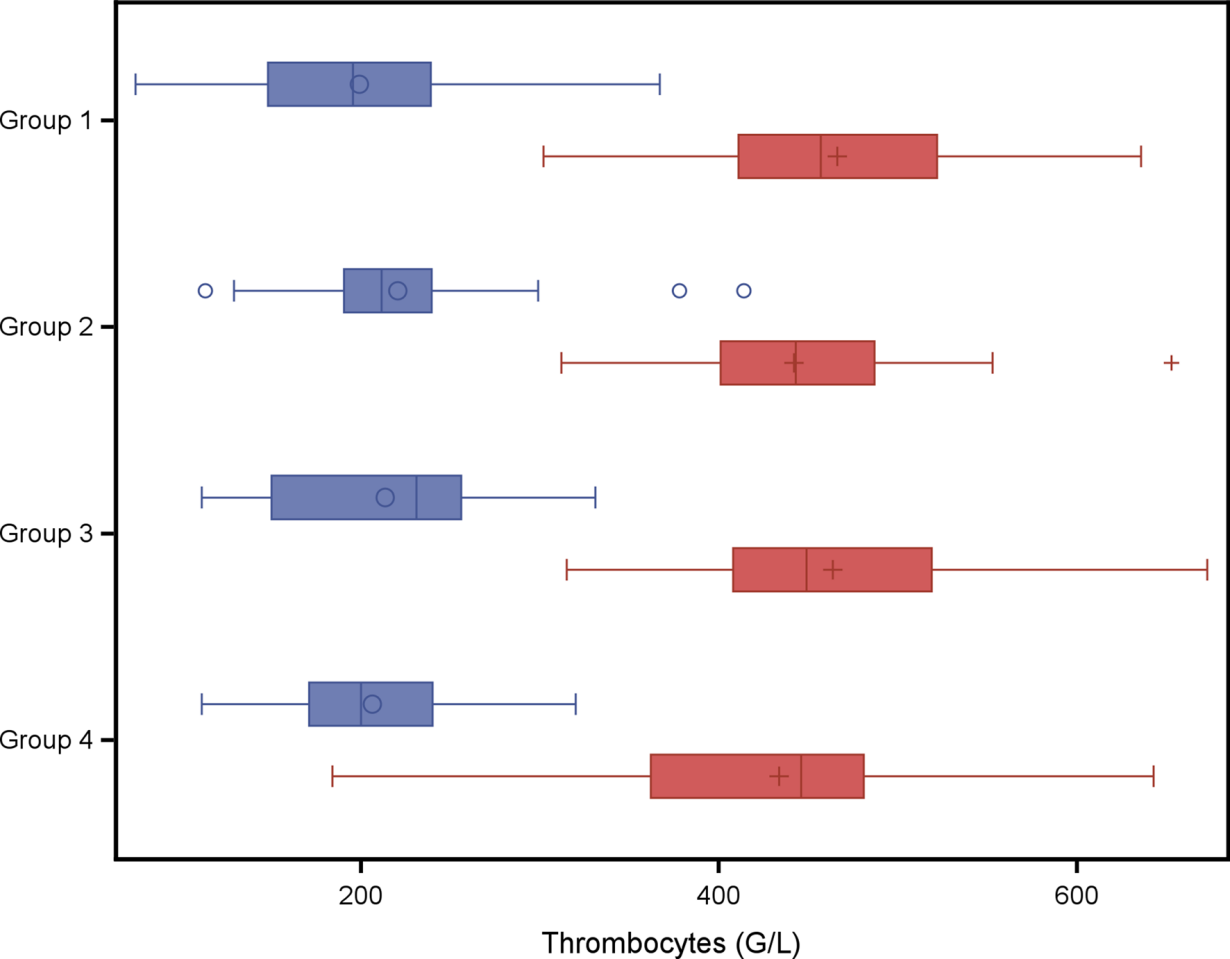
Thrombocytes (G/L) in piglets belonging to different treatment groups and day of life. Blue boxes: 2nd day of life, study start, red boxes: 24th day of life at weaning

On day 2 of life 33 pigs (25%) and at weaning only 2 pigs showed Hb values below 90 g/L. Both anaemic piglets at weaning suffered either from purulent navel inflammation or an umbilical hernia. Blood cell parameters are visualized in Figs. 1, 2, 3, 4 and 5 and summarized in Table 7. Manifold correlations between red blood cell parameters were found (data not shown). Hb at weaning showed a significant correlation with body weight at weaning (*P* < 0.01) and the ADWG in the suckling period (*P* = 0.002). The PCV at weaning was significantly correlated with weight at weaning, at end of nursery, at end of fattening and ADWG (*P* < 0.01). MCHC at weaning was significantly negatively correlated with weight at weaning, at end of nursery, at end of fattening and ADWG (*P* < 0.01).

**Table 7:** Mean (±standard deviation) haematological measures in different groups on day 2 and 24 of life

	Day of life	Group 1 (*n* = 33)	Group 2 (*n* = 34)	Group 3 (*n* = 31)	Group 4 (*n* = 33)
Haemoglobin (g/L)	2	95.4 (±11.1)	95.2 (±11.9)	98.4 (±8.5)	97.7 (±11.5)
	24	120.6 (±8.5)	121.8 (±11.6)	123.4 (±13.3)	126.3 (±11.1)
Packed cell volume (L/L)	2	0.28 (±0.03)	0.30 (±0.11)	0.29 (±0.03)	0.29 (±0.04)
	24	0.38 (±0.03)	0.38 (±0.04)	0.38 (±0.04)	0.39 (±0.04)
Mean corpuscular haemoglobin concentration (g/L)	2	335 (±11)	331 (±42)	339 (±15)	338 (±23)
	24	321 (±11)	323 (±12)	326 (±11)	325 (±10)
Red blood cell count (10^12^/L)	2	4.53 (±0.65)	4.42 (±0.52)	4.61 (±0.47)	4.55 (±0.661)
	24	5.92 (±0.45)	5.78 (±0.51)	5.88 (±0.52)	5.93 (±0.44)
Thrombocyte count (10^9^/L)	2	199 (±68)	221 (±61)	214 (±65)	206 (±50)
	24	466 (±85)	442 (±72)	464 (±87)	434 (±107)
Leucocyte count (10^9^/L)	2	8.05 (±1.77)	8.23 (±2.17)	8.09 (±1.83)	8.02 (±1.75)
	24	13.93 (±4.47)	14.11 (±4.67)	13.88 (±3.55)	14.33 (±5.17)

### Comparing treatment groups – equivalence and explorative comparisons

To underline the equivalence of treatments a non-inferiority test was conducted (TOST) as described. Based on statistical results shown in Table 8 the non-inferiority testing resulted in a P-value of 0.008, so that the first hypothesis, that iron dextran is non inferior to gleptoferron with respect to Hb at weaning was confirmed.

**Table 8:** Descriptive outcome of non-inferiority testing (TOST) with regard to haemoglobin concentrations (g/L) at weaning

Group	*N*	Mean (±standard deviation)	Mean 95% confidence limits	
1	33	120.6 (±8.5)	117.6	123.7
2	34	121.8 (±11.6)	117.8	125.8
Difference group 1–2 (Satterthwaite)		-1.16	-6.12	3.79

In a second explorative analysis the four treatment groups were compared with respect to red blood cell variables and weight gain. The analysis of variance models with fixed effect group did not result in any statistical significant group effect on variables listed in Tables 6 and 7. A group wise least square means comparison resulted in a significantly higher Hb concentration at weaning (*P* = 0.04) in piglets of group 4 (twice administration of 200 mg iron dextran on day 2 and day 11) compared to piglets of group 1 (one administration of iron dextran on day 2) as shown in Fig. 1. No significant group effect was found for PCV (Fig. 2), erythrocytes (Fig. 3), leucocytes (Fig. 4) or thrombocytes (Fig. 5).

**Fig. 6:**
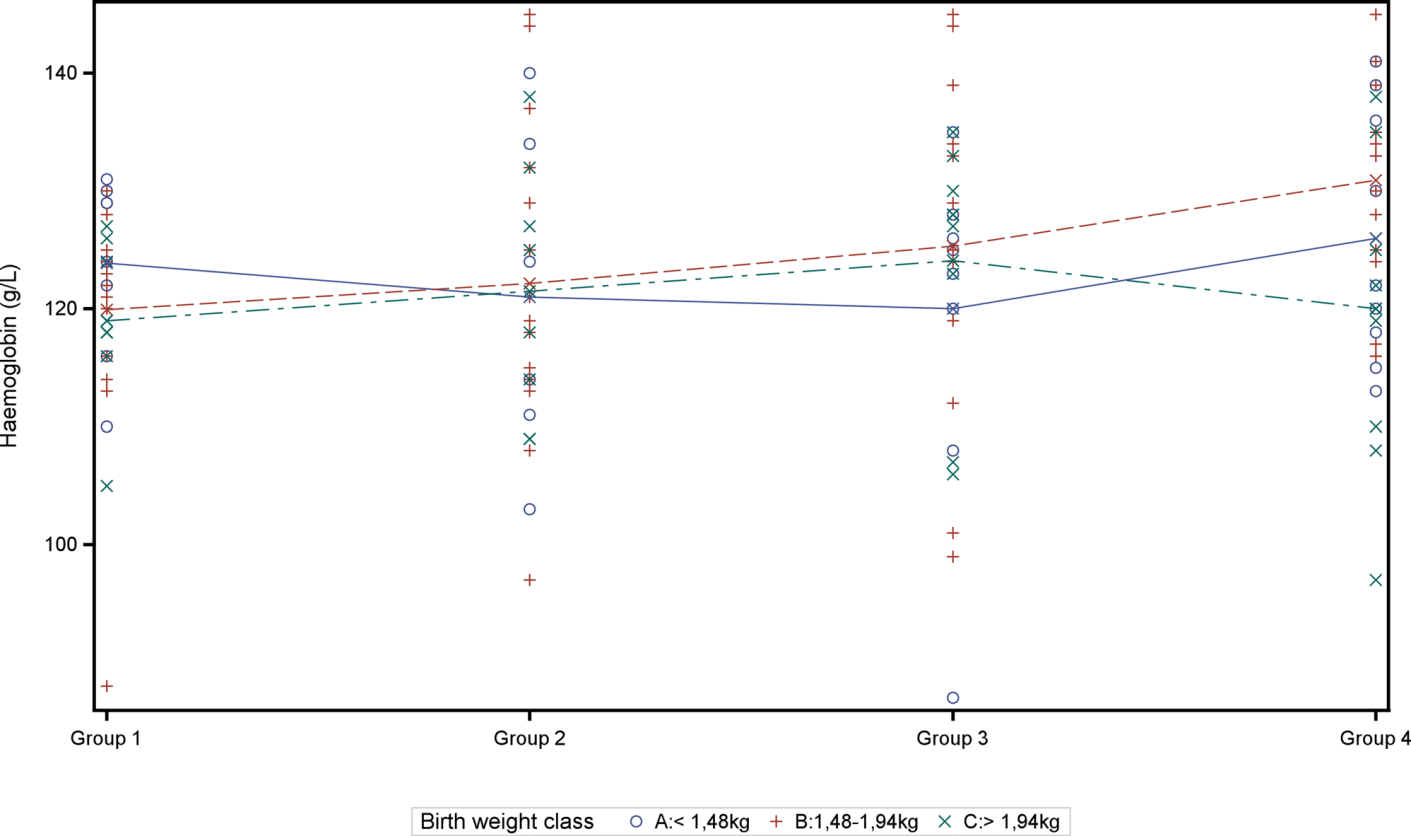
Interaction plot of different birth weight classes and treatment groups with effect on Hb concentrations. Haemoglobin (Hb) concentrations of individual piglets at weaning are depicted by symbols of the respective birth weight classes. Coloured lines indicate the mean Hb concentrations of the different birth weight classes in the different treatment groups at weaning

**Fig. 7:**
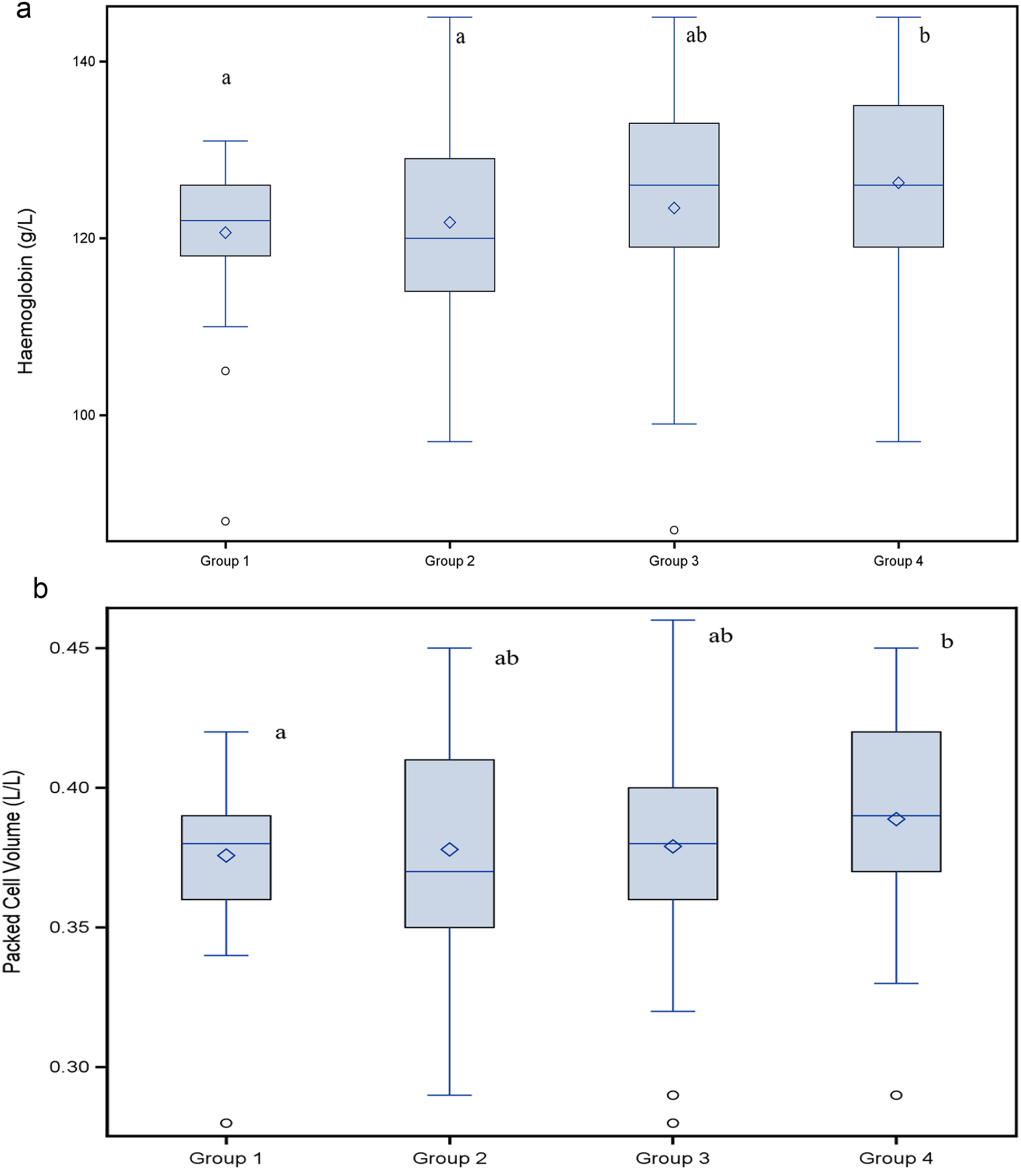
**(a)** Haemoglobin concentration (g/L) and **(b)** Packed cell volume (L/L) at weaning in medium weight piglets Medium weight piglets (1.48–1.94 kg body weight at study start) of group 1 and 4 differed in Hb concentration and PCV. Different letters right to the boxes indicate significant differences between respective groups

Finally, data exploration targeted to reveal different treatment effects in different birth weight classes of piglets. The two-way analysis of variance for the dependent variable Hb at weaning with fixed effects treatment group and weight class revealed no statistically significant effects. Visualization in an interaction plot hints towards countervailing effects as of Hb was affected in different ways by different treatments within the different weight classes (Fig. 6). While lowest and highest birth weight classes represent 25% of data each including extreme values, the median birth weight group represents the interquartile range and therefore 50% of the data without extreme values. In this subgroup a treatment response becomes visible with higher Hb concentrations in pigs treated on day 2 and day 11 of life with a full dose of iron dextran (Fig. 7a). In these medium weight piglets (interquartile weights at study start) significant differences in treatment effects on Hb at weaning were found between group 1 and 4 (*P* = 0.01) as well as between group 2 and 4 (*P* = 0.03). A significant difference in PCV at weaning was found between group 1 and 4 (*P* = 0.02). These findings reflect an effect of the repeated iron treatment on day 11 (Fig. 7b).

## Discussion

Iron (III)-hydroxide-dextran complex (Uniferon^®^, Pharmacosmos A/S, Holbaek, Denmark) is an injectable 20% iron dextran for prevention and treatment of anaemia in swine. In spite of the standard dose of 200 mg iron administered routinely to piglets within the first days of life, IDA can develop due to the high growth rates of modern crossbred pigs and especially in heavy pigs [10, 12, 35]. Next to anaemia, iron deficiency can negatively impact organ functions as especially the intestinal absorption and digestion capacities, due to e.g. destruction of tight junctions and decrease in villus height [36]. The benefit of a second iron dose was therefore examined in several studies, mostly resulting in increased Hb concentrations at weaning, while contradictory results were found for an effect on ADWG [7]. This might be due to known detrimental effects of routine iron bolus administrations, which can be of significance for piglet performance under certain factor constellations. Some authors therefore see no advantage in an additional iron dosage later in the suckling period [5, 7]. Several studies have focused on toxic effects of iron and the beneficial effects of split iron dosages to maintain the tightly balanced physiological iron metabolism [37]. Hepcidin increased significantly after intramuscular administration of 150 mg Fe/kg BW on day three of life, which was not shown with lower doses of 35.5 mg Fe/kg BW given twice on day three and 14 of life [38]. High hepcidin concentrations can block ferroportin with the consequence of lower iron utilization from external sources as creep feed or soil [39]. In one study, the administration of less iron in the first days of life (40 mg) was adequate to improve iron status of piglets without severely increasing the hepcidin expression. A split iron supplementation in piglets was therefore recommended to minimize DNA damage and oxidative stress [5]. These manifold different study results indicate that different protocols in iron administration might be suitable. Next to genetically defined growth intensity real iron requirements for piglets are dependent on several other farm-specific factors as e.g. blood loss during zootechnical measures, iron sources in the environment and health status of piglets. Therefor suitable protocols in iron administration might differ between farms, groups and individuals [32]. The exact need for iron supplementation is considered to be farm specific due to various factors influencing iron availability and requirements, so that diagnosis of IDA should be based on laboratory diagnostical findings [7]. Any parenteral iron supplementation should always target at a balance between the benefit of anaemia prevention and risk related to oxidative damage. These two sides of iron supplementation in piglets are still controversially discussed [36].

In this study, flexible iron dosages and two iron sources (iron dextran and gleptoferron) were compared. Iron dextran was non-inferior to gleptoferron in a combinatory product with respect to Hb concentrations at weaning. Following the non-inferiority concept, if there are benefits in dosage flexibility and costs - while the efficacy is not worse (non-inferior) than gleptoferron in a combinatory product - iron dextran is a reasonable and effective treatment. In the past, comparative studies between both iron sources came to different results. In a study with piglets out of 26 litters no differences in red blood parameters or ADWG were found between pigs treated with iron dextran or gleptoferron [40]. In two recent comparative studies with piglets from only four litters each, differences in various parameters were recorded, which were difficult to interpret due to a lack of sample size justification. In the first study, a comparison between the administration of 200 mg gleptoferron and 200 mg iron dextran in 25 piglets resulted in significant differences in mean Hb concentrations at weaning of 113 g/L in piglets treated with gleptoferron and 101 g/L in piglets treated with iron dextran [33]. Comparable to our study on day 2 of life 28% of the piglets had Hb concentrations < 90 g/L. On day 18 Hb values were all above 90 g/L and higher than at weaning on day 31, which was 7 days later than in our study. In the second study with 32 piglets mean Hb concentrations of 114 g/L at weaning did not differ between piglets treated with 200 mg gleptoferron or iron dextran, while mean concentration-time profiles of iron in serum were significantly increased in the gleptoferron group [34]. From serum iron levels no beneficial effect of iron administrations for the piglets can be deduced, because any bolus iron injection is reflected by increased tissue hepcidin expression as the systemic iron-regulatory hormone [37]. In case that more iron is supplemented than can be bound by transferrin in serum, free toxic iron can catalyze reactive oxygen species [38]. The intracellular iron storage protein ferritin in serum was also compared between the treatment groups but can also be interpreted as an inflammation marker due to its function as an acute phase protein [41]. For this reason, in our study Hb was defined as the target variable. Study design and number of pigs examined in our study differed from those in the cited comparative studies. No differences in ADWG were observed in the cited comparative studies, which is in accordance with our study. A higher dosage of iron on day 2 of life or a second full dosage of iron on day 11 of life had unexpectedly minor effects in our study. A second iron dose led to higher Hb concentrations but not ADWG at weaning. This outcome was similar to other studies, as e.g. in a comparison of gleptoferron injections at different points of time and oral iron supplementation, which led to different Hb concentrations but not ADWG between pigs with one or two iron injections [32]. A second iron dosage of 100 mg on day 11 of life did neither improve red blood cell parameters nor ADWG at weaning on day 21 in a study testing different iron dosages [42]. In contrast to that, in a recent study in organic pig production piglets profited clearly from a second dose of iron on day 14 of life due to the longer suckling period and subsequently later iron intake from feed [22].

If ADWG can be increased by additional administration of iron is still a matter of debate and might depend on several factors, as availability of iron from the sows, blood loss during zootechnical measures, start and iron content of creep feed. Different studies come to different results as reviewed previously [7]. A dose-dependent effect of iron dosage on ADWG in the suckling period was found up to a dosage of 100 mg iron on day 3 after birth and for up to 200 mg in the nursery period [42]. In most studies, no effect of an additional iron administration next to a primary dose of 200 mg on ADWG in the suckling period was found [43–45]. In some studies, the effect of a second iron dosage in the suckling period was only significant on ADWG in the post-weaning period [46]. This is not in accordance with findings in our study. In the post-weaning period several pigs suffered from *S.-suis*-related disease and in a later time period pig health was impacted by a tail-biting outbreak. Both diseases lead to inflammatory responses with influence on growth rates [47, 48]. Piglets with different birth weights and treated with the same dose of iron responded differently to iron provided in the nursery diet [46] and the effect of a second iron injection was also influenced by red blood cell parameters at the time point of second iron injection [43]. It can be hypothesized, that the significant effect of the second iron administration in medium weight piglets in our study might be due to a different Hb concentration at the respective time point in this group. Although pigs of different weight classes did not differ in their red blood cell parameters on their second day of life, differences can be expected on day 11 of life. Iron supply by only one injection in low weight piglets with lower ADWG might had been adequate, while high weight piglets might have profited from a second dose of iron resulting in higher Hb concentrations in the post-weaning period not examined in this study. For the medium weight piglets the beneficial effect of a second iron dose on Hb concentration was already detectable at weaning. In general, a positive association between ADWG in the post-weaning period and the Hb concentration at weaning exists [49]. Also in our study several red blood cell parameters were positively correlated with the ADWG.

In a recent study, a second iron injection resulted in a 4% increase in ADWG from weaning to slaughter [50]. In that study, piglets had no access to creep feed during the suckling period and diets contained less iron (100 mg/kg) than diets in our study (Table 1). Mean Hb concentrations at weaning in pigs with only one iron injection (107 g/L) were lower than in our study (group 1 and 2: 121 ± 10 g/L), where piglets had access to external iron sources in milk supplement and creep feed. In our study, iron content in creep feed was below 240 mg/kg as a published recommendation [7], but was within the range of iron concentrations in published creep feed studies [51, 52]. Creep feed containing 175 mg iron/kg and fed from day 7 after birth was found to be adequate for good growth rates irrespective of parenteral iron administration [28].

Voluntary iron intake by creep feed with appropriate iron content is considered an important iron source during the suckling period allowing iron absorption precisely regulated according to the needs of the piglets [4, 53, 54]. On the other hand, the regulation of intestinal iron absorption is not fully functional before 5–6 weeks of age [46]. Physiologically restricted iron absorption in the gut in young piglets is a protective mechanism to avoid injury of the intestinal barrier and negative influence on the gut microbiota favouring growth of pathogenic bacteria [36]. Any disturbance of gut development and gut flora, which can be due to a lack of colostrum especially in large litters, can have severe consequences for piglets’ health, especially in presence of pathogenic microorganisms. As pathogens require iron for their metabolism, any iron supplementation can interfere with the iron regulation of the host based on internalization of iron in intracellular compartments during inflammation [55]. In our study group the average numbers of live born and weaned piglets were in the upper range of piglet producing farms with high herd productivity in Europe [56]. Large litters need intensive care in the suckling period, usually including cross-fostering, which was not performed in this study [57]. The high proportion of pigs with head and carpal joint lesions in our study indicated increased teat fighting although milk supplement was provided. This finally did not negatively impact the suckling piglet mortality in the litters of the study group, so that management of these large litters can be assessed as successful in this farm. Nevertheless, the ADWG of 226 ± 69 g in the study pigs during the suckling period was relatively low [58]. Next to birth weight as the most important factor various factors related to sow, pen, litter and piglet have impact on ADWG in suckling piglets [59]. It was reported that foreleg lesions in piglets can affect weight gain in piglets. In our study carpal joint lesions were 37% in the second week of life, which is due to intense teat stimulation in the large litters with high competition. It can be assumed, that more cross fostering resulting in no more than one piglet per functional teat in a litter would have led to less carpal joint lesions and a higher ADWG. In three herds with ADWG in piglets ranging from 204 to 210 g sow-related factors had a significant impact on ADWG, as e.g. poorly milking sows three weeks after farrowing [59]. These sow-related factors were not analysed in our study in detail, but all sows were always in a good health status. *S. suis*-related disease was responsible for 7.5% piglet mortality in the nursery period, so that an inadequate colostrum supply due to the large litter sizes might have been a potential cofactor for the detrimental course of disease. Skin lesions at the head and carpal joints in weaned piglets were additional risk factors for entry of *S. suis* and subsequent bacteriaemia. A post-weaning ADWG of 475 ± 132 g in surviving pigs was within a physiological range [60].

To find a compromise between benefits and drawbacks of additional iron supplementation, new and flexible iron supply strategies are necessary taking different pig breeds, husbandry and management conditions into account. The advantage of combinatory products addressing anaemia and coccidiosis by one single shot can be counteracted by the lack of adjustment of the necessary amount of iron to prevent anaemia as long as possible. Additional scientific data are necessary to reassess iron supplementation strategies in pigs and other livestock animals with respect to the double-edged character of this element.

## Conclusions

In large litters of hyperprolific sows, a high heterogeneity of piglet birth weights is accompanied by a high growth potential resulting in varying demands for iron between individuals. While some individuals might benefit from 200 mg Fe, heavier piglets might be undersupplied, and smaller piglets might be burdened by this amount of iron due to its toxic effects. In our study iron dextran as a traditional substance was non-inferior to gleptoferron in a combinatory product. Iron dextran can be used in flexible dosage and treatment schemes according to varying requirements on different farms as an alternative product in case that no treatment against coccidiosis is necessary. In the study farm, Hb was increased at weaning by a second full dose of iron on day 11 of life, but without improvement of ADWG. Differences in published study outcomes about the effects of additional iron dosages might in part depend on piglet`s access to creep feed containing iron already in the suckling period superimposing the effect of additional parenteral iron administration. The consequences of metabolic changes by iron administration balancing its benefits should be addressed in further clinical studies comparing different protocols of iron administration.

## Data Availability

The data were collected by the scientific partners which provided written consent with the understanding that data would not be transferred to any third party. Therefore, data transfer to interested persons is not allowed without an additional formal contract. Data are available to qualified researchers who sign a contract with the University of Veterinary Medicine Hannover. This contract will include guarantees of the obligation to maintain data confidentially in accordance with the provisions of the European General Data Protection Regulation and its supporting documents in Germany. Currently, there is no data access committee or another body who could be contacted for the data. However, for this purpose, a committee will be founded. This future committee will consist of the authors, as well as members of the University of Veterinary Medicine Hannover and members of the funding partner. Interested cooperative partners who are able to sign a contract as described above may contact Isabel Hennig-Pauka (isabel.hennig-pauka@tiho-hannover.de), Field Station for Epidemiology, University of Veterinary Medicine Hannover, Buescheler Straße 9, 49456 Bakum.

## Declarations

### Prior publication

Data have not been published previously.
